# Incarcerated incisional hernia on an old orthopedics incision, a rare case report and a review of the literature

**DOI:** 10.1093/jscr/rjae369

**Published:** 2024-05-31

**Authors:** Mohammad Abu-Jeyyab, Mohammad Al-Jafari, Ibraheem M AlKhawaldeh, Sadeen Zein Eddin, Sophia Abu Tapanjeh, Mohannad Ja’Awin, Emad Aborajooh, Abdulqadir J Nashwan

**Affiliations:** Faculty of Medicine, Mutah University, Al-Karak 61710, Jordan; Red Crescent Hospital, Amman, Jordan; Faculty of Medicine, Mutah University, Al-Karak 61710, Jordan; Jameel Al-Totanji Hospital, Amman, Jordan; Faculty of Medicine, Mutah University, Al-Karak 61710, Jordan; Faculty of Medicine, Mutah University, Al-Karak 61710, Jordan; Faculty of Medicine, Mutah University, Al-Karak 61710, Jordan; Faculty of Medicine, Mutah University, Al-Karak 61710, Jordan; General Surgery and Anesthesia Department, Faculty of Medicine, Mutah University, Al-Karak 61710, Jordan; Nursing & Midwifery Research Department, Hamad Medical Corporation, Doha 3050, Qatar

**Keywords:** Incisional hernia, bowel obstruction, ORIF, distention

## Abstract

A previous surgical incision can lead to an abdominal wall defect known as an incisional hernia. The protrusion of abdominal viscera, particularly bowel loops, through this defect can result in various complications and affect organ function. Bowel loops are frequently involved and can lead to incarceration, obstruction or even strangulation. A 38-year-old male with a history of open reduction internal fixation for the left iliac wing presented with abdominal pain, vomiting and obstipation. Abdominal examination revealed a tender, distended abdominal area with swelling on the left hip. Radiological examination revealed bowel obstruction at the previous surgery site. During surgery, an incisional hernia was confirmed, and the bowel was found viable. Incisional hernias can occur even many years after primary surgery and may remain asymptomatic until complications arise. Elective hernial repair is recommended in some cases, such as the one presented here, as complications can be fatal.

## Introduction

An abdominal wall hernia, known as an incisional hernia, develops at the location of a prior surgical incision. Abdominal viscera, such as bowel loops, may protrude as a result of this defect; the protrusion of abdominal content may disrupt its function and cause a variety of problems. The bowel loop is one of the most common abdominal organs to protrude through abdominal defects, causing incarceration, obstruction or strangulation in some cases, with incarceration being the worst possible outcome if not surgically treated. The precise global epidemiology of incisional hernia is unknown. The wide variety of abdominal approaches, patient comorbidities and surgical methods for abdominal wall closure are all likely to contribute to a wide range of incidence rates that differ significantly between patient groups. An abdominal wall hernia, known as an incisional hernia, develops at the location of a prior surgical incision. Abdominal viscera, such as bowel loops, may protrude as a result of this defect; the protrusion of abdominal content may disrupt its function and cause a variety of problems. The bowel loop is one of the most common abdominal organs to protrude through abdominal defects, causing incarceration, obstruction or strangulation in some cases, with incarceration being the worst possible outcome if not surgically treated. The precise global epidemiology of incisional hernia is unknown. The wide variety of abdominal approaches, patient comorbidities and surgical methods for abdominal wall closure are all likely to contribute to a wide range of incidence rates that differ significantly between patient groups. According to meta-analyses of data from various countries, the incidence of incisional hernia ranges from 4 to 10%, depending on the type of operation. Thus far, no epidemiological research has been carried out [1]. Most incisional hernias are caused by abdominal procedures, as the rate of incisional hernia after laparotomies is 5–20%, and in high-risk patients, it is more than 30% [2]. These hernias seem to occur commonly after flank surgeries with an approximate incidence of ~17% [3]. It also arises after surgery in various locations but is regarded unusual, such as Incisional Hernia Following Minimally Invasive Lateral Retroperitoneal Surgery [4], kidney transplant incision [5], after a cesarean section [6], Appendix-associated incisional hernias [7], after a hip graft [8]. Incisional hernia repair includes using a synthetic mesh and can be done conventionally (open) or minimally (laparoscopically) [9, 10]. Here we present a rare case of a 38-year-old male patient with an Incarcerated incisional hernia on an old orthopedics incision.

## Case presentation

A 38-years-old male patient with a background of previous ORIF on the left side, and no other medical history presented to the emergency room with constipation, vomiting and abdominal pain. The ORIF was performed 15 years prior to the patient’s presentation following a left iliac wing fracture due to an RTA.

**Figure 1:**
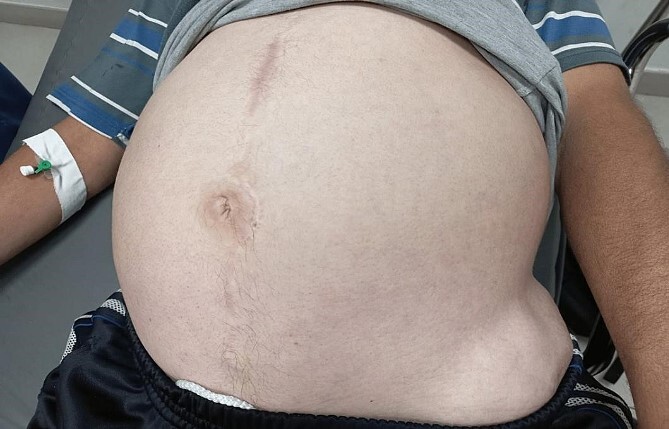
The patient’s abdomen at admission A.

**Figure 2:**
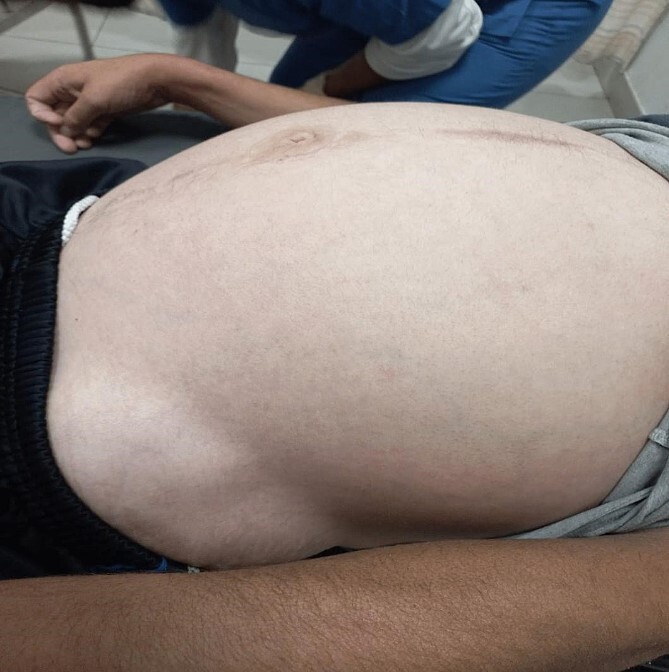
The patient’s abdomen at admission B.

By physical examination, the patient was found to have a distended and tender abdomen along with swelling and tenderness over the left hip. The patient was investigated and found to have a bowel obstruction. The patient was later found to have an incisional hernia at the place of the prior ORIF which required surgical intervention. Below is the inspection of the patient’s abdomen at admission (Figs 1 and 2).

### Radiodiagnosis imaging

**Figure 3:**
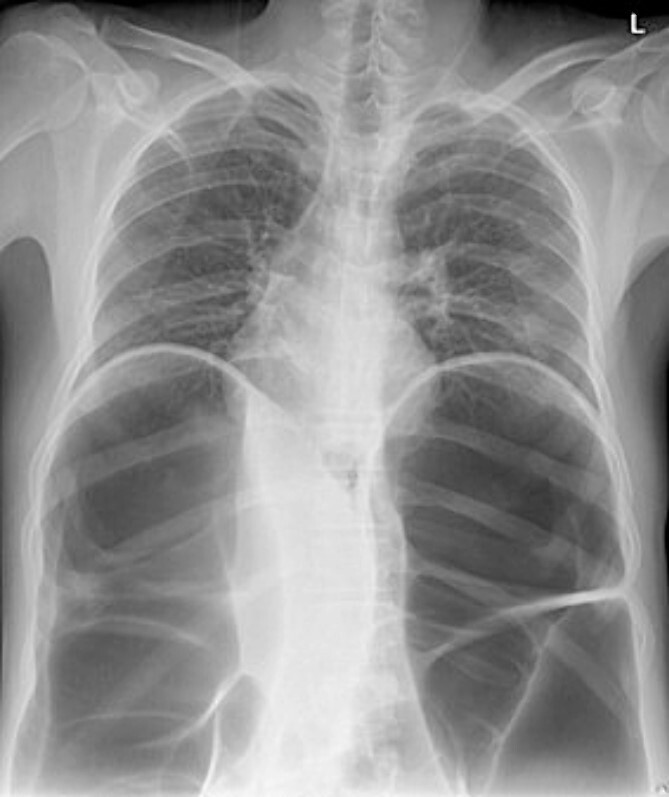
Chest X-ray.

**Figure 4:**
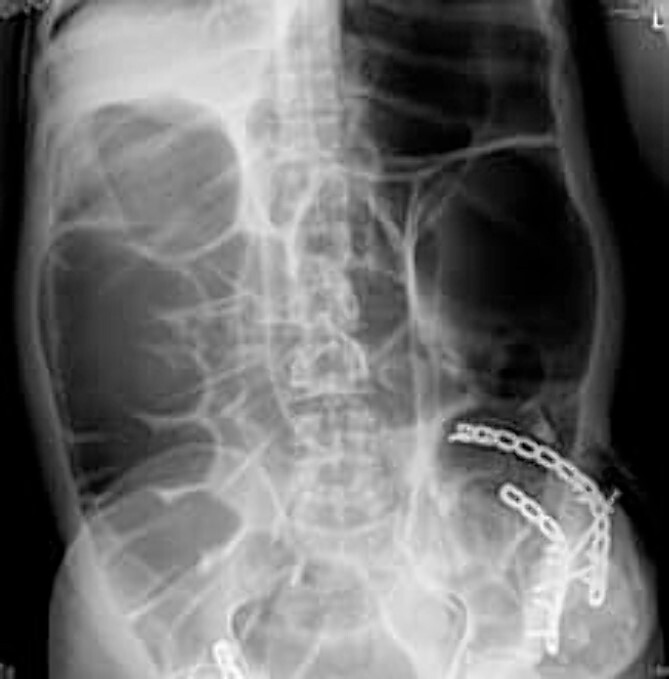
Erect abdomen X-ray.

Radio imaging was requested for the patient at admission. Erect X.R. has been taken, showing dilated bowel loops (Figs 3 and 4).

**Figure 5:**
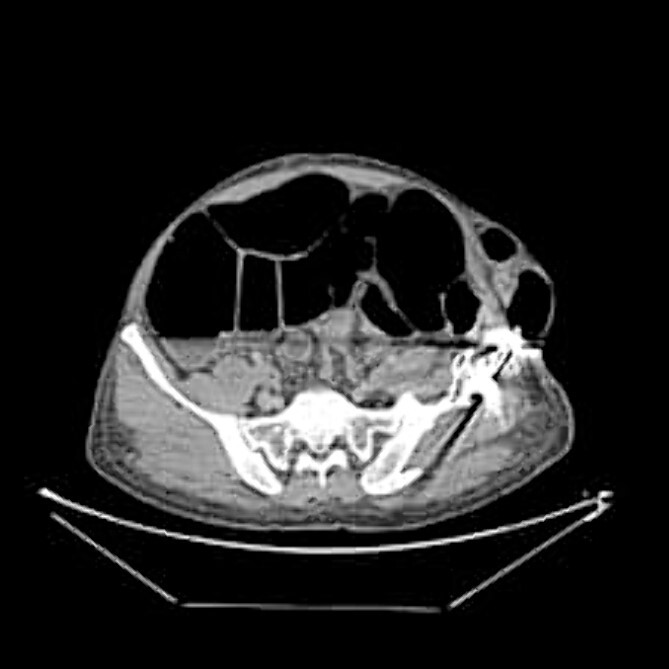
Axial abdominal CT scan.

**Figure 6:**
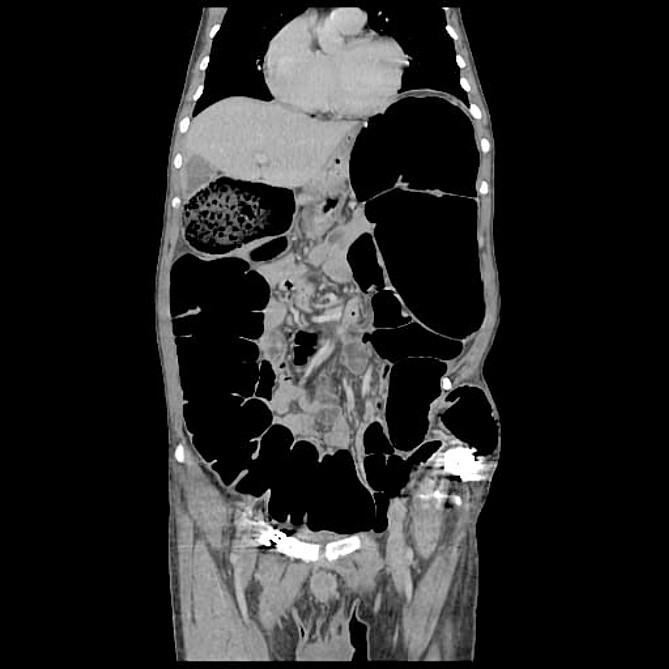
Corona l abdominal CT scan.

The CT abdomen pelvis (Figs 5 and 6) showed signs of bowel obstruction signs, along with an internal hernia at the place of the prior ORIF surgery. These findings were thought to be the core cause of the patient’s presentation in align with the taken history, physical examination of the patient.

### Surgical intervention

Given the presentation, the patient required surgical intervention. Intraoperatively, an internal incisional hernia was confirmed. During the procedure, the sigmoid and small bowel had to be removed from the hernia site. The hernia was repaired by applying mesh to the lateral abdominal wall. Decompression through the appendix was not successful as it was obliterated, and appendectomy was also performed. Furthermore, an enterotomy was required for suction and decompression, and the bowels were discovered to be viable.

### Post-operative period

The patient was hemodynamically stable, with normal results of blood tests. He was observed in the hospital and discharged on the fourth day post-op.

## Discussion

An unstable hemipelvis results from ilium fractures that break the continuity of the pelvic ring. Iliac fractures are more prevalent in the posterior section of the ilium, where the bone is the weakest. Unstable iliac fractures differ from pure iliac fractures that do not interfere with pelvic ring stability. Ilium fractures may extend into the sacroiliac joint; however, these fracture forms are discussed elsewhere. The acetabulum module describes iliac fractures that include the acetabulum [11].

The anterior approach to the iliac wing surgery and sacroiliac joint is appropriate for surgical exposure to manage the Dislocation of the sacroiliac joint as well as the Ilium fractures that extend into the sacroiliac joint. In the 1940s, Smith-Peterson described the direct anterior approach to the hip, and Hunter modified it in the 1950s. The muscle-saving nature of its interneurons intervals, earlier restoration of gait kinematics and lower dislocation rates are cited as advantages by proponents of this approach. This approach also provides for direct visualization of the anterior and superior sacroiliac joints. The direct anterior approach can be used with or without a dedicated table or fluoroscopy. Incisions are made along the iliac crest. Lastly, the aponeurosis of the external oblique muscle is carefully connected to the periosteum that was left attached laterally to the iliac crest after the incision is closed [9, 11, 12].

The muscle-preserving properties of the anterior technique have been linked to lower blood loss, faster functional recovery, lower dislocation rates and shorter hospital stays [13].

The following are some of the drawbacks of this strategy. The surgeon must first understand the anatomy of the pelvis and acetabulum. The lateral femoral cutaneous nerve travels laterally in the sartorius and can be injured if the surgeon is not careful when separating the sartorius/inguinal ligament from the ASIS. If the osteotomy is not completed, an iatrogenic fracture may occur. The tensor fascia lata and ASIS should be properly reconnected to the sartorius/inguinal ligament and external abdominal oblique aponeurosis. The probability of further issues is unknown [14].

Patients receiving an anterior approach to the iliac wing ORIF may develop hernias from a defect in the abdominal wall muscles. They must thus be regarded as real incisional hernias. Rather than lumbar hernias, despite the fact that their placement corresponds to the Petit triangle, where lumbar congenital hernias occur [15, 16].

These hernias are typically asymptomatic. However, when they occur, pain is the extremely crucial symptom. Incarceration and strangulation are exceedingly uncommon [17].

An incisional hernia is a kind of abdominal wall hernia that arises around a previous surgical incision. This is known as a ventral hernia. Incisional hernias in the midline are more common than those seen elsewhere. It might be a hernia with all of the typical hernia components of defect, sac, and content. There might also be a wall weakening with a shallow sac and the rare fluid bulging. It’s a common surgical problem. Incisional hernias are routinely evaluated by surgeons since they might cause symptoms in patients. The conventional look is a bulge with a positive cough impulse at the site of the incision. Complications from incisions. Patients with incisional hernias are also at a risk of incarceration, blockage or strangulation [18, 19].

Incisional hernias can form after any abdominal surgical procedure in which the abdominal wall is incised. After significant abdominal wall injuries, an incisional hernia has also been described. When the abdominal wall fails to close properly, incisional hernias occur [19, 20].

A growing soft-tissue mass at the region of the iliac defect and auscultation of bowel sounds above the hernia are early signs of herniation. Recurrent intraabdominal pressure increases promote progressive enlargement [21]. The patient in this report presented to the emergency department with symptoms of intestinal obstruction.

The preferred imaging method is computed tomography. While ultrasound is a low-cost, low-risk alternative for assessing these hernias, it does not reveal concomitant abdominal illness and is used less frequently in clinical settings [22, 23]. Conventional X-rays revealed dilated bowel segments. The computed tomography (CT) picture indicated several dilated colonic segments as well as a hernial defect on the left side of the abdomen. Moreover, ultrasonography of the abdomen and pelvis revealed a moderate reactive free fluid accumulation in the pelvis and a bowel loop imprisoned in the apparent bulge.

The surgical treatment of incisional hernias improves patients’ quality of life dramatically [9]. Three techniques for repairing incisional hernias are regularly used and should be adjusted to the patient and hernia features [24]. According to studies, there were various downsides to laparoscopic incisional hernia repair, including longer operating times. The price of providing equipment and specialist Skills and tools were used [25]. Yet, research has shown that in terms of short-term outcomes such as blood loss, hospital stay and early return to work, laparoscopic incisional hernia repair outperforms open hernia surgery [26]. In the discussed case, the used approach was open laparotomy, adhesiolysis, appendectomy and decomposition failure, then enterotomy decomposition was done and, finally, a Mesh was inserted, and the wound was closed.

## Conclusion

The discussed case is extremely rare. The case’s novelty is that it is a true hernia in an orthopedic surgery wound of the iliac wing ORIF, the clinical presentation and the surgical treatment used. There are not enough data about the epidemiology, prognosis or treatment guidelines. Reviewing the previous literature, we did not find any article describing a similar condition.

## Data Availability

All data generated or analysed during this study are included in this published article.
